# High pulse pressure is associated with an increased risk of diabetes in females but not in males: a retrospective cohort study

**DOI:** 10.1186/s13293-022-00482-8

**Published:** 2022-12-19

**Authors:** Sheng Jia, Xinyue Wang, Qing Yao, Jian Gao

**Affiliations:** 1 Department of Nutrition, Zhongshan Hospital (Xiamen Branch), Fudan University, Xiamen, 361015 Fujian China; 2 Department of Nutrition, Zhongshan Hospital, Fudan University, Shanghai, 200032 China

**Keywords:** Pulse pressure, Diabetes care, Sex differences

## Abstract

**Objective:**

Accumulating evidence suggests a close relationship between metabolic disturbance and increased arterial stiffness. However, whether there is an association between pulse pressure (PP) and diabetes and how this association might be impacted by sex is not clear.

**Methods:**

A total of 209,635 adult Chinese individuals > 20 years old across 32 sites and 11 cities in China (Shanghai, Beijing, Nanjing, Suzhou, Shenzhen, Changzhou, Chengdu, Guangzhou, Hefei, Wuhan, Nantong) were included in the study; participants were free of diabetes at baseline. In the present study, we analyzed the relationship between PP at baseline and incident diabetes using the Cox proportional hazard model.

**Results:**

During a median follow-up of 2.99 years, a total of 3971 participants (2885 men and 1086 women) developed diabetes, and the incidence was 6.3 per 1000 person-years. With each 10 mmHg increase in PP, the multivariable adjusted hazard ratio (HR) (95% confidence interval) for incident diabetes was 1.117 (1.061, 1.176) in females and 0.981 (0.951, 1.012) in males. Using the lowest quartile of PP as the reference category, the hazard ratio (HR) (95% CI) of the highest quartile of PP for incident diabetes was 1.494 (1.225, 1.822) in females and 0.939 (0.843, 1.045) in males. Smooth plots revealed a significant difference between males and females in the HRs for new-onset diabetes according to PP.

**Conclusion:**

Higher PP was related to future diabetes development in females but not in males and further research is needed to explore the mechanism.

**Supplementary Information:**

The online version contains supplementary material available at 10.1186/s13293-022-00482-8.

## Highlights

1. Elevated PP was associated with an increased risk of diabetes in females but not in males according to the multiple Cox regression model.
2. Sex differences in the association between PP and diabetes risk are not significantly affected by age.

**Supplementary Information:**

The online version contains supplementary material available at 10.1186/s13293-022-00482-8.

## Introduction

Blood pressure is an important indicator that reflects the status of circulation and is closely related to the occurrence of cardiovascular diseases. At the same time, higher blood pressure is a well-recognized risk factor for diabetes [1–3], but most studies focused only on systolic blood pressure (SBP) and diastolic blood pressure (DBP) rather than PP. PP represents the pulsatile component of blood pressure, and is defined as the difference in SBP and DBP that arises as a consequence of the episodic nature of cardiac contraction and the properties of arterial circulation [4].

The PP value is affected by a variety of cardiovascular factors (cardiac output, arterial elasticity, reflected waves, etc.), of which the arterial stiffness is the main factor. Therefore, PP is a well-recognized indirect measure of arterial stiffness that has been demonstrated to be associated with many cardiovascular events [4–6], and studies have shown that increased arterial stiffness may play a role in impaired glucose metabolism, metabolic syndrome and insulin resistance [7–9]. However, whether there is exact link between PP and diabetes risk has not been conclusively determined. Although there have been several relevant studies, the association of PP and diabetes risk is still in dispute. The studies by Rodrigo and Roland et al. reported that higher PP was associated with increased diabetes risk in patients after kidney transplantation, but the effect varied with follow-up time and sample size [10, 11]. In high-risk hypertensive patients, Yasuno et al. demonstrated that PP was a predictor of new-onset diabetes in high-risk (with SBP/DBP ≥ 180/110 mmHg or proteinuria or renal dysfunction or other comorbidities) hypertensive patients, and the increased risk was independent of other possible risk factors (such as body mass index (BMI), age and sex) [12]. Zhang et al. found that high PP was related to the incidence of type 2 diabetes mellitus (T2DM) in initially healthy, middle-aged females especially those with PP from 70 to 76 mm Hg and an age 52 to 59 years [13]. However, Liu et al. found that PP could not be an indicator of the risk of diabetes in Chinese middle-aged community residents with or without hypertension at baseline [14]. Considering the heterogeneity of the above studies in regard to population characteristics, that there are different blood pressure trends between males and females and that the sex differences are affected by age, we hypothesized that the relationship between PP and the risk of diabetes may be influenced by sex. Additionally, we conducted a further stratified analysis in the different age groups.

Therefore, the aim of this study, based on a large cohort of 209,635 participants across 32 locations in 11 cities in China, was to explore the potential association of PP and future diabetes risk in different sex groups and determine whether age plays a role in this association.

## Methods

### Study design and data source

This retrospective cohort study was based on a computerized database established by the Rich Healthcare Group in China, namely, the ‘DATADRYAD’ database (www.Datadryad.org). We downloaded the raw data for free from the site, provided by Chen et al. [15] from: association of body mass index and age with incident diabetes in Chinese adults: a population-based cohort study. (Dryad Digital Repository. 10.1136/bmjopen-2018-021768). The original study enrolled a total of 685,277 Chinese persons ≥ 20 years old who attended at least two visits from 2010 to 2016 across 32 sites and 11 cities in China (Shanghai, Beijing, Nanjing, Suzhou, Shenzhen, Changzhou, Chengdu, Guangzhou, Hefei, Wuhan, Nantong). The time of cohort entry was defined according to the date of the initial visit. At each visit to the health check center, participants completed a detailed questionnaire assessing demographic, lifestyle and family history of chronic disease. The trained staff administered the clinical measurements, including measurements of body weight, height and blood pressure. Fasting venous blood samples were collected after a fast of at least a 10 h at each visit. Plasma glucose levels were measured by the glucose oxidase method on an autoanalyzer (Beckman 5800). BMI was equal to the weight divided by the square of height. The data were collected under standardized conditions in accordance with uniform procedures. Laboratory methods were also carefully standardized through stringent internal and external quality controls.

The authors of the original study waived all copyright and related ownership of the raw data. Therefore, we could use these data for secondary analysis without infringing on the authors’ rights. Furthermore, the original study was approved by the Rich Healthcare Group Review Board, and the information was retrieved retrospectively. The original study was conducted in accordance with the Declaration of Helsinki, as was this secondary research. The data were anonymous, and the requirement for informed consent was waived by the Rich Healthcare Group Review Board due to the observational nature of the study.

### Study sample

**Fig. 1:**
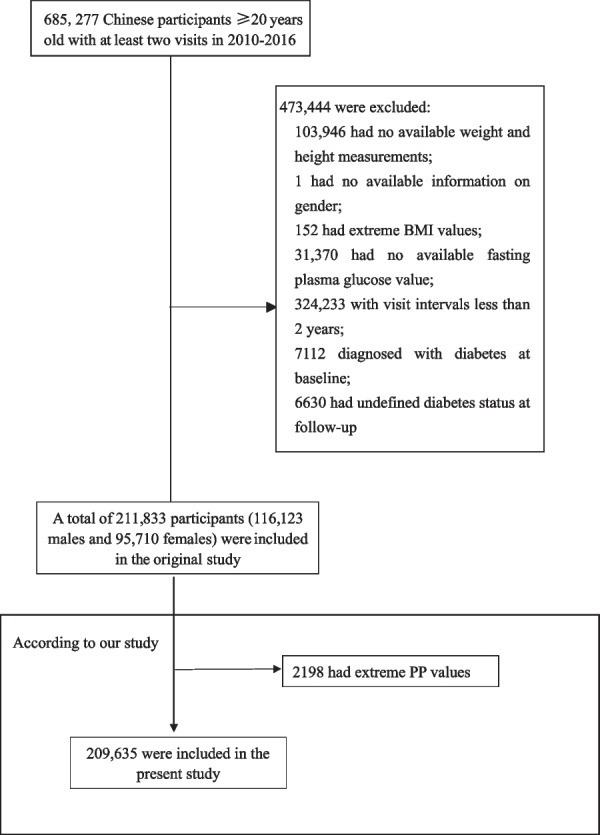
Flowchart of study participants

Consistent with the original study, participants aged 20–99 years who attended at least two visits between 2010 and 2016 were eligible for inclusion in our research. Participants were excluded at baseline in the original study if they met any of the following criteria: (1) no available information on weight, height or sex; (2) extreme BMI values (< 15 kg/m^2^ or > 55 kg/m^2^); (3) visit intervals < 2 years; (4) no available fasting plasma glucose values; and (5) diagnosis of diabetes at baseline or undefined diabetes status at follow-up. A total of 211,833 participants remained after applying the exclusion criteria in the original study [15]. In the present study, we further excluded participants with incomplete blood pressure and extreme PP values (mean ± 3 standard deviations, *n* = 2198). Figure 1 depicts the participant selection process. Finally, our study included 209,635 participants in the secondary analysis.

### Exposure and outcome measures

The outcome of interest was incident diabetes. Diabetes mellitus was defined as fasting plasma glucose ≥ 7.00 mmol/L and/or self-reported diabetes during the follow-up period [15]. Patients were censored at the time of diagnosis of diabetes or the last visit, whichever came first.

The exposure of interest was PP which was defined as the difference between SBP and DBP. Blood pressure values were obtained by trained staff using standard mercury sphygmomanometers through office blood pressure measurements. Covariates of interest included age, sex, BMI, fasting plasma glucose (FPG), smoking status, alcohol consumption status, and family history of diabetes.

### Statistical analyses

Continuous variables are expressed as the means ± standard deviations (normal distribution) or medians (quartiles) (skewed distribution), and categorical variables are expressed as frequency or percentages. Missing values for each categorical covariate (smoking and alcohol consumption status) are considered as a group.

A multiple Cox regression model was used to explore the association between PP at baseline and diabetes risk, expressed as HRs with 95% CI, which were calculated both for each 10 mmHg increase in PP and across the quartiles of PP. Covariates in the multivariable models included age, BMI, baseline FPG, smoking status, alcohol consumption status and family history of diabetes. All BP measures were not included simultaneously in regression analysis to avoid any collinearity that these independent variables may have. Sensitivity analysis was carried out in different models after excluding participants with missing values. The *E*-value was calculated to evaluate unmeasured confounding, which is defined as the minimum strength of association on the risk ratio scale, that an unmeasured confounder would need to have with both the treatment and the outcome to fully explain a specific treatment–outcome association, conditional on the measured covariates [16]. Multivariate adjusted smooth curve fitting was used to explore sex differences in the association between PP and diabetes risk (expressed as log RR for incident diabetes). A *P* value ≤ 0.05 was considered statistically significant. All statistical analyses were performed with SPSS 25.0 (IBM SPSS Inc, Chicago, IL) and R version 3.5.3.

## Results

### Baseline characteristics of the study participants

In the present study, we identified 209,635 participants (54.8% men and 45.2% women) who met our inclusion criteria (a flowchart of the study participants is shown in Fig. 1). The mean age of the sample was 42 ± 12 years. The mean PP was 44 ± 11 mmHg (male: 46 ± 12 mmHg, female: 43 ± 12 mmHg). During the median follow-up of 3 years, a total of 3971 participants (2885 men and 1086 women) developed diabetes. The crude incidence of diabetes was 18.9 per 1000 person-years. The baseline clinical and biochemical characteristics of the participants are presented in Table 1.

**Table 1:** Clinical and biochemical characteristics of the study cohort at baseline

Variables	All (*n* = 209,635)		Male (*n* = 114,972)	Female (*n* = 94,663)	*P*
Age (years)	42 ± 12		42 ± 13	42 ± 12	< 0.001
BMI (kg/m^2^)	23.22 ± 3.34		24.18 ± 3.25	22.09 ± 3.08	< 0.001
SBP (mm Hg)	119 ± 16		123 ± 15	114 ± 16	< 0.001
DBP (mm Hg)	74 ± 11		77 ± 11	71 ± 10	< 0.001
PP (mm Hg)	44 ± 11		46 ± 12	43 ± 12	< 0.001
FPG (mmol/L)	4.91 ± 0.61		4.98 ± 0.63	4.84 ± 0.58	< 0.001
Year of follow-up (years)	2.99 (2.16–3.95)		3.00 (2.16–3.95)	2.99 (2.16–3.95)	< 0.001
Smoking status (%)					< 0.001
Current smoker	5.7		10.4	0.02	
Ever smoker	1.2		2.22	0.03	
Never smoker	21.5		21.2	21.9	
Not recorded	71.5		66.2	78.0	
Alcohol consumption status (%)					< 0.001
Current drinker	0.6		1.14	0.03	
Ever drinker	4.3		7.28	0.58	
Never drinker	23.6		25.4	21.4	
Not recorded	71.5		66.2	78.0	
Family history of diabetes (%)					< 0.001
No	97.9		98.6	97.2	
Yes	2.1		1.4	2.8	

*BMI* body mass index, *SBP* systolic blood pressure, *DBP* diastolic blood pressure, *PP* pulse pressure, *FPG* fasting plasma glucose

### Association between PP and incident diabetes

As shown in Table 2, the risk of incident diabetes increased with higher PP (per 10 mm Hg) according to the univariate Cox regression analysis both in males (HR: 1.247, 95% CI: 1.207,1.288) and females (HR: 1.963, 95% CI: 1.873,2.058). However, after further adjusting for potential confounders, the significance in males was diminished (HR: 0.981, 95%CI: 0.951,1.012), but not that in females (HR: 1.117, 95% CI: 1.061,1.176). There were similar associations between quartiles of PP and incident diabetes in males and females. Elevated PP was associated with an increased risk of diabetes in females comparing the highest PP category with the lowest category (HR: 1.494, 95% CI: 1.225,1.822), but this relationship was not significant in males (HR: 0.939, 95% CI: 0.843,1.045). Sensitivity analysis was performed in the participants with no missing values (male = 38,898, female = 20,811), which was consistent with the previous results (Additional file 1: Table S1). In addition, the results did not change significantly after further stratification by age (Table 3). Higher PP was not significantly associated with diabetes risk in males among those with an age < 50 years or those with an age ≥ 50 years, and the association in females remained significant in both age groups. We also did Cox regression analyses for the associations between SBP, DBP, BMI and diabetes risk, and no sex differences were found (Additional file 1: Table S2). The results of an analysis that assessed potential unmeasured confounding are provided in Additional file 1: Table S3 (*E*-value: 1.49, *E*-value upper limit: 1.31).

**Table 2:** Relationship between PP and incident diabetes in different models by sex

PP (mm Hg)	Male (*n* = 114,972), HR (95% CI)			Female (*n* = 94,663), HR (95% CI)		
	Unadjusted	Model 1	Model 2	Unadjusted	Model 1	Model 2
Per 10 mmHg	1.247 (1.207,1.288)	1.056 (1.023,1.090)	0.981 (0.951,1.012)	1.963 (1.873,2.058)	1.248 (1.185,1.315)	1.117 (1.061,1.176)
1st quartile (< 37 mmHg)	Reference	Reference	Reference	Reference	Reference	Reference
2nd quartile (37–43 mmHg)	1.032 (0.912,1.169)	1.030 (0.909,1.166)	0.988 (0.873,1.119)	1.353 (1.071,1.708)	1.147 (0.908,1.450)	1.064 (0.842,1.344)
3rd quartile (44-51 mmHg)	1.185 (1.059,1.327)	1.150 (1.027,1.288)	1.031 (0.921,1.155)	2.493 (2.026,3.068)	1.701 (1.379,2.097)	1.466 (1.189,1.807)
4th quartile (≥ 51 mmHg)	1.636 (1.472,1.818)	1.173 (1.053,1.306)	0.939 (0.843,1.045)	6.533 (5.417,7.879)	2.122 (1.736,2.595)	1.494 (1.225,1.822)
*P* for trend	< 0.0001	0.007	0.246	< 0.0001	< 0.0001	< 0.0001

Model 1: Adjusted for age, BMI, smoking, alcohol consumption and family history of diabetes

Model 2: Adjusted for age, BMI, smoking, alcohol consumption, family history of diabetes and FPG

**Table 3:** Relationships between PP and incident diabetes stratified by sex and age

PP (mm Hg)	Male (*n* = 114,972)		Female (*n* = 94663)	
	HR, age < 50 (*n* = 84,305)	HR, age ≥ 50 (*n* = 30,667)	HR, age < 50 (*n* = 72,329)	HR, age ≥ 50 (*n* = 22,334)
Per 10 mmHg for PP	0.952 (0.900,1.007)	1.037 (0.997,1.078)	1.132 (1.014,1.263)	1.116 (1.053,1.183)
1st quartile (< 37 mmHg)	Reference	Reference	Reference	Reference
2nd quartile (37–44 mm Hg)	0.818 (0.675,0.991)	1.152 (0.977,1.357)	0.719 (0.503,1.027)	1.060 (0.771,1.458)
3rd quartile (44–51 mm Hg)	0.878 (0.740,1.043)	1.224 (1.051,1.425)	1.433 (1.063,1.933)	1.371 (1.035,1.816)
4th quartile (≥ 51 mm Hg)	0.829 (0.697,0.986)	1.158 (1.004,1.335)	1.481 (1.081,2.030)	1.487 (1.150,1.922)
*P* for trend	0.128	0.074	< 0.001	0.003

### Multivariate adjusted smooth curve fitting of log RR for incident diabetes and PP stratified by sex

**Fig. 2:**
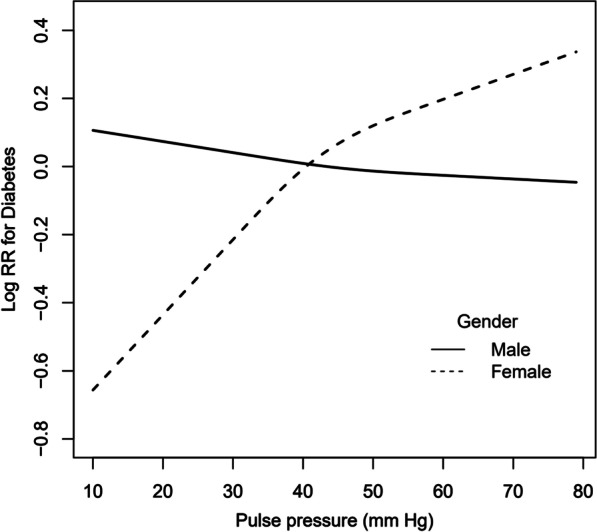
Multivariate adjusted smooth curve fitting of log RR for incident diabetes and PP stratified by sex. The model was adjusted for age, BMI, smoking, alcohol consumption, family history of diabetes and FPG

As shown in Fig. 2, smooth curve fitting of log RR for incident diabetes and PP stratified by sex also showed the significantly different associations of PP with diabetes risk between males and females. The log RR for incident diabetes was obviously increased as PP increased in females, while the curve was relatively flat in males, which suggested a weak association.

## Discussion

To date, few studies have focused on PP and diabetes risk, and the conclusions were controversial. To our knowledge, this is the first study that has examined the associations of PP with the risk of diabetes in such a large general population. In the present study, we showed that higher PP was significantly correlated with an increased risk of diabetes in females but not in males after adjusting for age, BMI, baseline FPG, family history of diabetes, smoking and alcohol consumption status. This finding suggests that increased PP may be related to the development of new-onset diabetes in females, although the mechanism of this association remains to be elucidated.

An early study on PP and diabetes risk in hypertensive patients reported by Yasuno et al. showed that high PP is an independent risk factor for diabetes in hypertensive patients [11]. However, it was not initially designed to prospectively evaluate this association and it was a post hoc analysis. Moreover, whether new-onset diabetes had occurred simply depended on the participating investigators’ reports. Therefore, the conclusions on PP and diabetes risk are not reliable. Different from the present study, another cohort study that focused on the middle-aged community population from China did not find an association between PP and diabetes risk [14]. However, the study included a very small sample (*n* = 687), which decreased the reliability of the study. The same limitation was present in Janghorbani’s study [17], in which they also did not find a significant association between PP and diabetes risk in nondiabetic first-degree relatives of patients with T2DM (*n* = 701). Another Chinese cohort study reported that a significant association between high PP (PP > 60 mmHg) and the risk of diabetes was found only in women aged 52 to 59 years, but not in older or younger women, which has not been reasonably explained. This may be related to the low statistical power due to the small sample size after multiple groupings. Considering that there is a progressive increase in SBP with aging for adults, while DBP tends to remain constant or decline after the fifth to sixth decade; as a consequence, PP increases progressively with age and the rate of rise accelerates after age 50 years [18] and that the age of menopause is around 50 years, analysis stratified by age (< 50 or ≥ 50 years) was further conducted. In this regard, this study still found an increased risk of diabetes in women with high pulse pressure in both age groups, further indicating that the potential impact of sex on the association between PP and diabetes risk may not be affected by age. Further research is needed to explain the role of sex.

The mechanism underlying the association between high PP and diabetes risk possibly relates to arteriosclerosis which can lead to microvascular dysfunction [19]. Previous studies have demonstrated that microcirculation dysfunction is related to impaired tissue perfusion, which can increase the risk of diabetes by impairing insulin-mediated changes in muscle perfusion and glucose metabolism [20, 21]. On the other hand, arterial stiffness can lead to high blood pressure, and hypertension is also a well-recognized risk factor for diabetes. The differences in the risk of diabetes between males and females suggest that sex hormones may play a role in the association of PP with the pathogenesis of diabetes. Importantly, although our age-stratified analysis yielded similar findings for women < 50 or ≥ 50 years, this group stratification was based solely on age and not menopausal status, making it difficult to discern the potential role of estrogen. On the other hand, the sex differences observed may also be due to differences in stature between males and females (females are typically shorter). However, adjusting for height in the Cox regression model did not significantly change our results (data not shown), suggesting that stature may not be driving the sex differences. To our knowledge, there has been no relevant study to explain the results, and further research is warranted.

Some limitations of this study warrant mentioning. First, as with any observational study, there is potential for residual confounding, but the present study has the largest sample size among previous studies, and we calculated unmeasured confounding to adjust our results. The second limitation was the method of diabetes diagnosis. Considering the large sample size and feasibility, the original study did not diagnose diabetes using oral glucose tolerance test or HbA1c, which meant that the incidence of diabetes may be underestimated. However, there were studies that have shown a linear relationship between HbA1c and FPG [22, 23], and the large sample size in this study can also reduce this limitation to some extent. Third, due to the large study population, there was inevitably a certain number of missing values, for which we have considered the missing values for each categorical covariate as a separate group in the analysis. Finally, because the study included mainly individuals who underwent active physical examinations in China, the generalizability of our findings to other ethnic groups or general populations may be limited. However, the sample of the study consisted of a wide range of apparently healthy adults and the data came from several sites in China. The results of the present study can be generalized to a wider Chinese population.

### Perspectives and significance

Although there have been many studies on the association of blood pressure with diabetes, this study is the first to report sex differences in the association of PP with diabetes risk in such a large sample. In this study, we demonstrated that higher PP was associated with an increased risk of diabetes only in females, independent of other possible risk factors for new-onset diabetes. The association of PP with diabetes risk may be due to arterial stiffness. However, the underlying mechanism of the sex differences remains to be elucidated. This study suggests that screening PP, especially in women, may help to identify patients who are at increased risk of diabetes and therefore aid in early therapeutic decision-making, and PP should also be considered in the treatment of hypertension.

## Supplementary Information

**Additional** **file** **1:** **Table** **S1.** Relationship between PP and incident diabetes in different models by sex after excluding participants with missing values. **Table** **S2.** Comparison of the associations for PP, SBP, DBP, BMI with diabetes risk using Cox regression analysis. **Table** **S3.** E-values indicating unmeasured confounding for incident diabetes in females.

## Data Availability

The datasets used during the current study are available in the Dryad Digital Repository, https://doi.org/10.5061/dryad.ft8750v.

## Abbreviations

PP: Pulse pressure
HR: Hazard ratio
SBP: Systolic blood pressure
DBP: Diastolic blood pressure
BMI: Body mass index
FPG: Fasting plasma glucose
