# Supplementary material for: High pulse pressure is associated with an increased risk of diabetes in females but not in males: a retrospective cohort study
Source: Biol Sex Differ. 2022 Dec 19;13:72. doi: 10.1186/s13293-022-00482-8 (PMC9764461; doi:10.1186/s13293-022-00482-8)
Supplement: Supplementary file 1 — Additional file 1: Table S1. Relationship between PP and incident diabetes in different models by sex after excluding participants with missing values. Table S2. Comparison of the associations for PP, SBP, DBP, BMI with diabetes risk using Cox regression analysis. Table S3. E-values indicating unmeasured confounding for incident diabetes in females. [file 13293_2022_482_MOESM1_ESM.docx]

# Additional file 1

**Table S1. Relationship between PP and incident diabetes in different models by sex after excluding participants with missing values.**

| **PP (mm Hg)** | **Male (n=38898), HR (95% CI)** | | |  | **Femal**e **(n=20811), HR (95% CI)** | | |
| --- | --- | --- | --- | --- | --- | --- | --- |
|  | Unadjusted | Model 1 | Model 2 |  | Unadjusted | Model 1 | Model 2 |
| per 10 mm Hg | 1.27 (1.198,1.347) | 1.051 (0.992,1.113) | 1.011 (0.956,1.07) |  | 2.099 (1.895,2.325) | 1.278 (1.142,1.43) | 1.176 (1.052,1.315) |
| 1st quartile (<37 mmHg) | Reference | Reference | Reference |  | Reference | Reference | Reference |
| 2nd quartile (37-43 mmHg) | 0.924 (0.738,1.155) | 0.942 (0.753,1.178) | 0.965 (0.771,1.208) |  | 1.652 (0.945,2.888) | 1.410(0.804,2.472) | 1.327 (0.757,2.325) |
| 3rd quartile (44-51mmHg) | 1.137 (0.931,1.387) | 1.106 (0.906,1.351) | 1.058 (0.867,1.293) |  | 3.102 (1.887,5.1) | 2.176 (1.319,3.591) | 1.926 (1.167,3.181) |
| 4th quartile (≥51 mmHg) | 1.646 (1.367,1.982) | 1.142 (0.943,1.382) | 1.00(0.827,1.208) |  | 8.716 (5.54,13.713) | 2.672 (1.657,4.31) | 2.081 (1.289,3.359) |
| P for trend | <0.001 | 0.207 | 0.835 |  | <0.0001 | <0.0001 | 0.010 |

Model 1: Adjusted for age, BMI, smoking, alcohol consumption and family history of diabetes;

Model 2: Adjusted for age, BMI, smoking, alcohol consumption, family history of diabetes and FPG

**Table S2: Comparison of the associations for PP, SBP, DBP, BMI with diabetes risk using Cox regression analysis.**

|  | Male, n=114972 | |  | Female, n=94663 | |
| --- | --- | --- | --- | --- | --- |
|  | HR | *P* |  | HR | *P* |
| Per 1 SD for PP | 1.002 (0.967, 1.039) | 0.911 |  | 1.176 (1.107, 1.250) | <0.0001 |
| Per 1 SD for SBP | 1.111 (1.073, 1.149) | <0.0001 |  | 1.289 (1.217, 1.365) | <0.0001 |
| Per 1 SD for DBP | 1.153 (1.115, 1.193) | <0.0001 |  | 1.187 (1.128, 1.249) | <0.0001 |
| Per 1 SD for BMI | 1.462 (1.412, 1.515) | <0.0001 |  | 1.385 (1.322, 1.451) | <0.0001 |
| The model was adjusted for age, FPG at baseline, smoking status, drinking status and family history of diabetes. | | | | | |

**Table S3: E-values indicating unmeasured confounding for incident diabetes in females.**

| Outcome | E-value | E-value Upper Limit |
| --- | --- | --- |
| Incident diabetes | 1.49 | 1.31 |

E-value interpretations: For incident diabetes, the reported hazard ratio could be explained away by an unmeasured confounder that was associated with both the exposure and the outcome by a risk ratio of 1.49-fold each.
